# The Effectiveness of Guided Imagery on Walking and Balance Dysfunction in Patients With Multiple Sclerosis: A Randomized Controlled Trial

**DOI:** 10.1155/bn/9961468

**Published:** 2025-04-21

**Authors:** Atena Shojaie, Hoda Kamali, Monir Sadat Nematollahi, Zahra Imani Goghary, Behnaz Bagherian

**Affiliations:** ^1^Department of Medical-Surgical Nursing, School of Nursing and Midwifery, Kerman University of Medical Sciences, Kerman, Iran; ^2^Neurology Research Center, Kerman University of Medical Sciences, Kerman, Iran; ^3^School of Nursing and Midwifery, Reproductive and Family Health Research Center, Kerman University of Medical Sciences, Kerman, Iran; ^4^Department of Nursing and Midwifery, Sirjan School of Medical Sciences, Kerman, Iran; ^5^Department of Medical-Surgical Nursing, School of Nursing and Midwifery, Nursing Research Center, Kerman University of Medical Sciences, Kerman, Iran

**Keywords:** balance, gait disorder, guided imagery, multiple sclerosis

## Abstract

**Background:** Multiple sclerosis can cause walking and balance disorders. These complications cause extensive disturbances in the quality of life, independence, and self-care and affect many aspects of their lives. The guided imagery method is a simple, easy, and safe intervention.

**Methods:** A randomized controlled trial was conducted to determine the effect of guided imagery on walking and balance dysfunction in patients with multiple sclerosis in 2023. Sixty-six patients were randomly divided into two intervention and control groups (*n* = 33). The intervention group listened to the audio file of nature-based guided imagery, and the control group did not receive any intervention. Data were collected by 6-min walk test, 25-ft walk test, and Berg balance scale before and after the intervention. Analysis of the average results of movement disorder and balance intra- and intergroup, before and after the study, was done with a paired *t*-test, independent *t*-test, and analysis of covariance. The significance level in all tests is 0.05, with a confidence level of 95%.

**Results:** After performing guided imagery, in the intervention group, walking dysfunction significantly improved, with changes from 429.55 ± 42.78 to 459.24 ± 41.48 in the 6-min walk test, 7.12 ± 0.37 to 6.74 ± 0.44 in the 25-ft walk test, and the improvement of balance level with changes of 26.60 ± 3.13 to 31.72 ± 3.64 in the Berg balance test.

**Conclusion:** Using guided imagery can improve walking dysfunction and balance in patients with multiple sclerosis.

**Trial Registration:** Iranian Registry of Clinical Trials: IRCT20220614055170N1

## 1. Introduction and Background

Multiple sclerosis (MS) is a chronic disorder of the central nervous system (CNS) with unknown etiology that is the most common cause of nontraumatic disability among young adults [1]. Currently, there are approximately 2.5 million patients with MS (PWMS) worldwide and 400,000 in the United States, with an average global prevalence of 33 per 100,000, which varies among countries [2].

Walking disorder is considered as the most limiting symptom on patients' quality of life [3]. Research results in Europe showed that one out of two PWMS reported movement disorders in the first month of diagnosis, and more than 90% of patients reported severe movement disorders within 10 years [4].

Balance disorder is also one of the most debilitating complications associated with MS, which affects about 75% of patients during the disease [5]. Imbalance is seen in most PWMS, even in the presence of minimal disability, because balance requires many of the main functions of the CNS that are affected by MS. [6] The most important complication of imbalance in these patients is the risk of falling. Statistics show that 93% of PWMS between the ages of 21 and 73 express their fear of falling due to imbalance, and 57% of these people have lost at least once during a 6-month follow-up [5].

Many pharmacological and nonpharmacological methods control movement and balance disorders in PWMS. The use of disease-modifying drugs has the effect of slowing the progression of disabilities, and less evidence supports their effectiveness in reversing motor disability and restoring motor function in PWMS. According to the evidence in the use of auditory–motor pairing, cognitive methods as simple, useful, and uncomplicated methods seem to be effective in improving the movement ability of these patients [7].

Several studies have been conducted in the field of reducing walking dysfunction and imbalance in PWMS. Moumdjian et al. maintained that listening to a musical file with specific beats in PWMS and synchronizing walking with the beats of the song could lead to a better and faster walking pattern and reduce mental fatigue [8]. Kahraman et al. improved walking speed, maintaining balance and quality of life of MS patients using telerehabilitation-based motor imagery training [9]. Mohaghegh et al. expressed that the use of rhythmic auditory stimulation during walking of PWMS can cause the auditory rhythm to be reflected in the motor output together with the auditory–motor synchrony in the brain–spinal network [10].

Among nonpharmacological interventions for the management of symptoms in PWMS, a simple and safe method is guided imagery. Guided imagery is a cognitive intervention in which a visual situation is simulated for the patient; it is a mental representation that occurs without the need for an external stimulus; in this process, the patients listen to the words of a guide (the real presence of a person or a recorded voice) and visualize a series of mental images in their mind and absorb them as a real image [11].

Guided imagery is one of the branches of psychosomatic medicine that helps the patient to do self-healing using inner forces [12]. Decety in 1996 stated that guided imagery activates points in the brain that are activated during the actual execution of movements; in other words, the brain is exercised in the absence of physical activity [13]. Although the main mechanism of the mental imagery process is not clear, reorganization of the cerebral cortex in response to visual stimuli likely increases the absorption of the sensory–motor cortex and, as a result, improves the motor performance of patients [14].

The result of using motor imagery in improving the walking pattern of patients with movement disorders indicates that the gait, speed, and symmetry of the patient's steps in the presence of the visualized auditory stimulus show a high level of improvement [15]. However, no study was found in which a nature-based guided imagery intervention was used to improve movement disorders. Since MS is a multifocal disease involving different physical and cognitive aspects of the patient, the present study was conducted to investigate the effect of guided imagery on walking and balance dysfunction in PWMS.

## 2. Materials and Methods

This research is a randomized clinical trial with two groups of intervention and control, which was conducted in 2023. The study was approved by the Research Council (IR.KMU.REC.1402.289).

**Figure 1:**
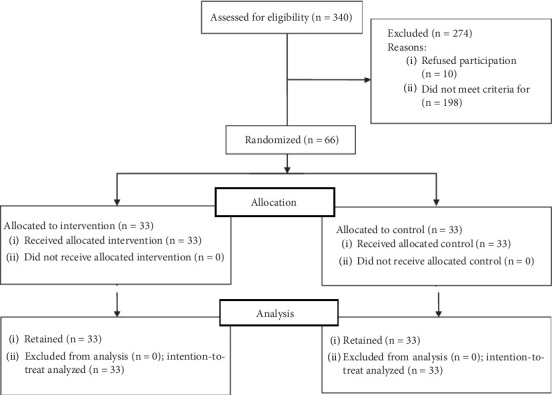
CONSORT map.

The sample size was computed by calculating the effect size (0.747) based on conducting a pilot study on 20 patients (without considering this number of samples in the total sample size). Using the specialized G-Power software, the sample size was calculated to be 33 patients in each group, considering 10% attrition. Available sampling was done among the patients referred to the desired treatment center. Out of the total population of 340 PWMS, randomly, 66 patients who fulfilled the inclusion criteria were included and divided into two intervention (*n* = 33) and control (*n* = 33) groups using the simple randomized method by computer. The intervention and control groups were matched in terms of the duration of MS before the intervention (Figure 1).

The inclusion criteria were as follows: having MS of relapsing-remitting MS (RRMS), primary progressive MS (PPMS), or secondary progressive MS (SPMS) with the confirmation and diagnosis of a neurologist; ability to communicate in Persian; acceptable speaking ability; acceptable hearing ability so that the patient can hear the researcher's voice and audio file completely and clearly; age range of 18–60 years [16]; ability to walk at least 50 m using the patient's usual mobility aids such as canes and walkers in a 6-min walk test (6MWT) before the intervention [17]; not suffering from any primary neurological, psychological, cardiac, respiratory, and other diseases such as osteoarthritis or rheumatoid arthritis and musculoskeletal diseases that affect the patient's usual movement ability; failure to participate in a rehabilitation program or physical activity that affects the disease in at least the last 2 months or changing the treatment in a way that affects the patient's movement ability (physiotherapy or drug treatment) [8]; and not having used high-dose steroids in the past month. High-dose steroid therapy is defined according to the following protocol: steroid administration of 50 mg/day intravenously (IV) for 6 days, or 40 mg intramuscularly (IM) for a longer period of 15 days, or 20 mg IM for more than 30 days [18]; not having experienced any severe clinical relapse in disease in the last month; receiving a score between 1 and 5.6 on the Extended Disability Status Scale (EDSS), which indicated mild to moderate disability [17]; and at least 1 year had passed since the diagnosis of MS. [8] The exclusion criteria were pregnancy [17, 19].

A personal and demographic questionnaire (including age, gender, marital status, educational status, height, weight, duration of MS, and type of MS) was completed before the study. The 6MWT, the 25-ft walk test (25FWT), and the Berg balance scale (BBS) were used to measure the primary outcomes. In the 6MWT, the ability of patients to walk on a flat path in 6 min was evaluated by determining the speed by themselves [20]. In the 25FWT, the patient was instructed to walk a 25-ft course as quickly and safely as possible with the endpoint marked by a red sign [21]. The BBS is a valid tool for measuring balance and the risk of falling in individuals of different ages and genders. It consists of 14 items, each scored on a scale of 0–4 [22].

The intervention in this study involved nature-based guided imagery delivered through the playback of audio-recorded content by the researcher. The audio file was verified by experts in this field and played through the Anker Sound Core Life 2 Neo headset with the ability to produce powerful and high-quality sound. The maximum content playback time was 15 min. The broadcasted file focused on depicting a view of nature in the form of natural landscapes with accompanying sounds suitable for the described landscape, while the smells, sounds, and sensations were reported in it.

The patients present in both groups underwent a single 6MWT and a 25FWT, and their balance was evaluated using the BBS. Before the tests, all patients were instructed to avoid consuming heavy meals, wearing restrictive clothing, smoking, drinking alcohol, and exercising. The navigable path in this research was a smooth 20-m course without obstacles, inclines, or declines.

The participants in the intervention group listened to the guided imagery audio file for 15 min, after which they performed three tests again. A 15-min rest period was provided between each test. The safety of all patients in the study was ensured by maintaining a safe environment and path (no obstacles and no vehicle traffic), as well as providing protection and care to patients while walking by the research team.

The control group repeated all three tests after a 15-min rest period, without listening to any audio file. Due to the inability to exchange information, the two groups were examined separately on different days but under the same conditions and at the same time of day.

## 3. Results

Of the total number of subjects (*N* = 66), most of the participants were male, were married, and had diplomas. Also, most of the patients had MS type of RRMS. The average duration of MS disease was in the range of 5–8 years. As shown in Table 1, there was no statistically significant difference between the two groups in terms of demographic characteristics (*p* > 0.05) (Table 1).

### 3.1. Within-Group Changes for Walking Dysfunction

The results revealed that the intervention group exhibited increased average scores on the 6MWT and decreased time on the 25FWT among PWMS after the intervention. According to the results presented in Table 2, the within-group changes in the intervention group demonstrated that the average score for walking impairment in the 6MWT increased from 429.55 ± 42.78 to 459.24 ± 41.48, indicating a significant increase of 29.68 units based on the results of the paired *t*-test (*p* < 0.001) (Table 2).

Furthermore, as shown in Table 3, the results of the 25FWT in the intervention group following the implementation of the intervention demonstrated changes within the group from 7.12 ± 0.37 to 6.74 ± 0.44, indicating a decrease of 0.37 units. According to the results of the paired *t*-test, this difference was found to be significant (*p* < 0.001) (Table 3).

Additionally, the results revealed that in the control group, the average scores on the 6MWT exhibited changes from 430.17 ± 41.23 to 429.09 ± 40.47, indicating a decrease of 1.08 units. However, based on the results of the *t*-test, this pairwise difference was not found to be significant (*p* > 0.05) (Table 2). Similarly, in the 25FWT, changes were observed from 6.90 ± 0.28 to 6.92 ± 0.26, equivalent to a change of 0.01 units. According to the results of the paired *t*-test, these changes were also not found to be significant (*p* > 0.05) (Table 3).

### 3.2. Intergroup Changes for Walking Dysfunction

The results of the independent *t*-test indicated that there was no significant difference in the average scores of the intervention and control groups on the 6MWT before the intervention (*p* = 0.952). Similarly, there was no statistically significant difference in the scores of the 25FWT between the intervention and control groups before the intervention (*p* = 0.097). Therefore, the two groups had similar walking disorder scores based on the 6MWT and 25FWT before the intervention.

A comparison was made between the changes in the 6MWT and the 25FWT in the intervention and control groups after the intervention. The results of the independent *t*-test indicated a significant difference between the scores before and after the intervention in both the 6MWT and the 25FWT (*p* < 0.001).

Furthermore, after the implementation of guided imagery, the results of the covariance analysis demonstrated a significant difference in the average scores for walking disorders based on the 6MWT and 25FWT between the intervention and control groups (*p* < 0.001). Specifically, the scores on the 6MWT had increased more in the intervention group compared to the control group, while the scores on the 25FWT had decreased more in the intervention group than in the control group. Based on the partial eta squared, the effect of guided imagery on the improvement of walking disorder, as measured by the 6MWT and the 25FWT, in PWMS was 71.5% and 30.4%, respectively (Tables 2 and 3).

### 3.3. Within-Group Changes for Imbalance

The results showed that in the intervention group, the average balance score in PWMS increased after the intervention. Thus, within-group changes showed that in the intervention group, the balance changed from 26.60 ± 3.13 to 31.72 ± 3.64, equal to an increase of 5.12 units compared to before the intervention. Based on the results of the paired *t*-test, a significant difference was observed in the balance variable score (*p* < 0.001) (Table 4).

Also, the results in the control group showed that the average balance did not change. As the intragroup changes showed, in the control group, the amount of balance changes was from 27.33 ± 2.38 to 26.93 ± 2.04, equal to a change of 0.39 units. Based on the results of the paired *t*-test, no significant difference was observed in the balance score in PWMS (*p* = 0.119) (Table 4).

### 3.4. Intergroup Changes for Imbalance

The results of the independent *t*-test showed that before the intervention, there was no statistically significant difference between the average balance score of PWMS in the intervention and control groups (*p* = 0.292). Therefore, the two groups were the same in terms of the balance score.

Furthermore, after the implementation of guided imagery, the results of the covariance analysis showed that there was a significant difference in the average balance score between the intervention and control groups (*p* < 0.001). Specifically, balance in the intervention group increased more than in the control group. Based on the partial eta squared, the effect of guided imagery on increasing balance in PWMS was 75% (Table 4).

## 4. Discussion

The main finding of the present study indicates that the average score of walking dysfunction in the intervention group, before and after the intervention, has significantly decreased according to the results of the 6MWT and the 25FWT, which indicates that guided imagery reduces the severity of walking dysfunction in PWMS.

This finding is not consistent with the study conducted by McLoughlin et al. In their study, they performed a 6MWT on 34 patients with moderate severity MS and showed that motor dysfunction in these patients during the 6MWT could be caused by fatigue resulting from reduced ankle flexion and decreased force absorption in the hip, knee, and ankle area. This movement disorder is not related to mental functions [23]. Fatigue caused by MS can manifest as both motor and nonmotor fatigue. Previous studies have confirmed the centrality of the thalamus in establishing a connection between the structures of the cerebral cortex and its role in causing fatigue in PWMS. Therefore, increasing the activity and communication within the thalamic network as demonstrated in the present study could reduce the transmission of motor neuron messages to the cerebral cortex and improve movement by stimulating auditory–motor connections [24].

The results of Henning et al.'s study in 2021, investigating the effect of talking while walking, on the walking pattern of PWMS, are consistent with the result of the present study. The findings of this study showed that even unrelated conversations such as counting numbers, and saying words that begin with a specific letter by activating new points of the cerebral cortex that are not normally active, cause a change in the activity of the motor cortex of the brain and the central system of the thalamus [25].

The consistent results of De Sanctis et al.'s study in 2020 showed that the dual cognitive–motor walking pattern in MS patients significantly improved the severity of movement disorders by using their brain's additional resources at no cost or an equivalent cost to their healthy peers [26].

Brambila-Tapia et al. examined psychoemotional changes in PWMS and showed that stressful events and psychoemotional patterns in these patients can act as an explosive or aggravating factor of the disease and this issue affects the severity of motor complications and quality of life. In this study, the use of cognitive methods such as guided imagery reduced mental and motor disorders in PWMS and led to an improvement in the patient's quality of life [27].

Mind–body-based interventions, including guided imagery, can reduce the production of the proinflammatory protein nuclear factor kappa B (NF-KB). This reduction can decrease the activation of the sympathetic nervous system and the production of inflammatory factors, thereby reducing psychological and motor symptoms and complications in patients [28]. Additionally, another study stated that the use of mental imagery makes changes in the hypothalamus–pituitary–adrenal axis [29]. These methods have the potential to provide treatment modality along with other current MS treatments, as explored in a 2018 study by Case et al. In this study, the researchers used light-guided imagery in PWMS, and the results showed that this method was effective in decreasing pain, chronic disability, depression, and fatigue and reducing physical symptoms in these patients [30].

The results of a meta-analysis by Kho et al., which studied six clinical trials, investigated the effect of guided imagery on improving upper limb movement in patients with hemiplegia after stroke. The results showed that guided imagery was an effective intervention to improve movement and neurological adaptation in stroke patients [31].

In the present study, the average score of balance in PWMS has increased significantly after guided imagery in the intervention group, which means that guided imagery is effective in improving the balance of PWMS.

The results of a systematic review by Corrini et al. in examining the effectiveness of different therapeutic approaches to improve balance in PWMS showed that dual cognitive–motor exercises and their repetition can increase neural flexibility and stimulate specific neural pathways to increase motor efficiency and also increase the number of synapses in the motor cortex [5].

Molhemi et al. stated that the chronic nature of MS and the repetitive, continuous nature of rehabilitation programs could reduce patients' participation. Therefore, more effective and enjoyable rehabilitation programs could help maintain patients' commitment and motivation [6]. The use of mental processes by creating a metacognition and providing multisensory feedback to patients can facilitate the movement process, maintain balance, and reduce the possibility of falling [32]. From the neurophysiological perspective, cognitive exercises can activate the mirror nervous system and, by transforming the visual–motor or auditory–motor phenomenon, create a motor action replica and store it in the CNS to serve as a model for real performance. Additionally, creating sensory feedback induces sensorimotor neuroplasticity in the cerebral cortex aiding in the restoration of balance function [33].

According to Jacobson's [34] psychoneuromuscular theory, mental activity is essentially a weakened physical activity. Mental exercises induce the activity and partial contraction of the muscles and the motor feedback resulting from these partial activities is sent to the brain centers. Consequently, this affects the activity of these areas leading to the strengthening and improvement in neuromuscular coordination of supporting and opposing muscles as well as auxiliary muscles [35].

Schmidt's activity-arousal theory also states that mental imagery causes an increase in the level of arousal and physiological activity and helps the person imagining to reach the threshold of performing the activity [36].

## 5. Conclusion

The results of this study showed that guided imagery is efficacious in improving walking and balance dysfunction in PWMS. Therefore, the use of cognitive and mental methods alongside medical treatments can help reduce the risks posed by the disease and, in the long term, improve the quality of life while reducing movement and balance disorders of PWMS.

## 6. Limitations

The limitations of this study were some physical conditions including fatigue, boredom, disabilities of some patients, or some environmental factors that could have affected the patients' level of attention and concentration on the audio file. The low sample size also reduced the generalizability of the findings.

## 7. Recommendations for Further Research

Since the present study was conducted on PWMS as a chronic disease, it is recommended to investigate the effect of similar interventions on improving movement disorders in other conditions such as muscular dystrophies, Guillain–Barré, spinal cord injuries, and even acute diseases.

## Figures and Tables

**Table 1:** Demographic data of the subjects.

**Demographic data**	**Intervention group (** **n** = 33**)**		**Control group (** **n** = 33**)**		**Test results**
	**Number**	**Percentage**	**Number**	**Percentage**	
Gender					
Male	18	54.5	17	51.5	*p* = 0.805 *χ*^2^(1) = 0.06
Female	15	45.5	16	48.5	
Marital status					
Single	2	6.1	0	0	*p* = 0.351 *χ*^2^(2) = 2.09
Married	25	75.8	26	78.8	
Others	6	18.1	7	21.2	
Educational status					
Under diploma	12	36.4	13	39.4	*p* = 0.965 *χ*^2^(2) = 0.07
Diploma	16	48.5	15	45.5	
Graduated	5	15.1	5	15.1	
Type of MS					
RRMS	20	60.6	27	81.8	*p* = 0.057 *χ*^2^(1) = 3.62
PPMS	13	39.4	6	18.2	
Age (years) (standard deviation) average	48.21 (7.51)		46.36 (7.10)		*p* = 0.308 *T*(64) = 1.03
Duration of MS (years) (standard deviation) average	6.30 (1.21)		6.36 (1.05)		*p* = 0.829 *T*(64) = −0.22
Body mass index (standard deviation) average	23.83 (3.01)		23.56 (2.47)		*p* = 0.690 *T*(64) = 0.40

*Note: χ* = chi-square test; *T* = independent *t*-test.

**Table 2:** The average score of walking dysfunction of patients with multiple sclerosis in two intervention and control groups in the 6-min walk test.

**Variable**	**Group**	**Before intervention** **(** **s** **t** **a** **n** **d** **a** **r** **d** **deviation** ± **mean)**	**After the intervention** **(** **s** **t** **a** **n** **d** **a** **r** **d** **deviation** ± **mean)**	**Average changes (confidence interval)**	**Intragroup statistical estimation**
6-min walk test	Intervention	429.55 ± 42.78	459.24 ± 42.78	−29.68 (−34.55 to −24.81)	*t* ^∗^ = −12.42 *p* < 0.001
	Control	430.17 ± 41.23	429.09 ± 40.47	1.08 (−0.31 to 2.49)	*t* ^∗^ = 1.58 *p* = 0.125
	Intergroup estimation	*t* ^∗∗^ = −0.06 *p* = 0.952	*F* ^∗∗∗^ = 158.06 *p* < 0.001 Partial eta squared = 0.715	*t* ^∗∗^ = −12.37 *p* < 0.001	

⁣^∗^Paired *t*-test. ⁣^∗∗^Independent *t*-test. ⁣^∗∗∗^Analysis of covariance test.

**Table 3:** The average score of walking dysfunction of patients with multiple sclerosis in two intervention and control groups in the 25-ft walk test.

**Variable**	**Group**	**Before intervention** **(** **s** **t** **a** **n** **d** **a** **r** **d** **deviation** ± **mean)**	**After the intervention** **(** **s** **t** **a** **n** **d** **a** **r** **d** **deviation** ± **mean)**	**Average changes (confidence interval)**	**Intragroup statistical estimation**
25-ft walk test	Intervention	7.12 ± 0.37	6.74 ± 0.44	−0.37 (−0.50 to −0.25)	*t* ^∗^ = −6.03 *p* < 0.001
	Control	6.90 ± 0.28	6.92 ± 0.26	−0.01 (−0.05 to 0.02)	*t* ^∗^ = −0.96 *p* = 0.342
	Intergroup estimation	*t* ^∗∗^ = −1.68 *p* = 0.097	*F* ^∗∗∗^ = 27.46 *p* < 0.001 Partial eta squared = 0.304	*t* ^∗∗^ = −5.49 *p* < 0.001	

⁣^∗^Paired *t*-test. ⁣^∗∗^Independent *t*-test. ⁣^∗∗∗^Analysis of covariance test.

**Table 4:** Average balance score of multiple sclerosis patients in two intervention and control groups.

**Variable**	**Group**	**Before intervention** **(** **s** **t** **a** **n** **d** **a** **r** **d** **deviation** ± **mean)**	**After the intervention** **(** **s** **t** **a** **n** **d** **a** **r** **d** **deviation** ± **mean)**	**Average changes (confidence interval)**	**Intragroup statistical estimation**
Balance	Intervention	26.60 ± 3.13	31.72 ± 3.64	−5.12 (−5.75 to −4.48)	*t* ^∗^ = −16.52 *p* < 0.001
	Control	27.33 ± 2.38	26.93 ± 2.04	0.39 (−0.10 to 0.89)	*t* ^∗^ = 1.60 *p* = 0.119
	Intergroup estimation	*t* ^∗∗^ = −1.06 *p* = 0.292	*F* ^∗∗∗^ = 188.80 *p* < 0.001 Partial eta squared = 0.750	*t* ^∗∗^ = −13.94 *p* < 0.001	

⁣^∗^Paired *t*-test. ⁣^∗∗^Independent *t*-test. ⁣^∗∗∗^Analysis of covariance test.

## Data Availability

The audio file would be available to both the control and intervention groups. The data of this research can be shared as public open data. Protocols, methods, and materials will be available. The audio file of guided imagery will also be made available to the public of patients, researchers, and other people who need to use it through communication with the researcher.
